# The Influence of Sunlight Exposure and Sun Protecting Behaviours on Allergic Outcomes in Early Childhood

**DOI:** 10.3390/ijerph18105429

**Published:** 2021-05-19

**Authors:** Kristina Rueter, Anderson P. Jones, Aris Siafarikas, Paola Chivers, Susan L. Prescott, Debra J. Palmer

**Affiliations:** 1 School of Medicine, The University of Western Australia, 35 Stirling Highway, Crawley 6009, Australia; kristina.rueter@health.wa.gov.au (K.R.); aris.siafarikas@health.wa.gov.au (A.S.); susan.prescott@telethonkids.org.au (S.L.P.); 2 Department of Immunology and Dermatology, Perth Children’s Hospital, 15 Hospital Avenue, Nedlands 6009, Australia; 3 inVIVO Planetary Health, Group of the Worldwide Universities Network (WUN), 6010 Park Ave, West New York, NJ 07093, USA; 4 Telethon Kids Institute, University of Western Australia, 15 Hospital Avenue, Nedlands 6009, Australia; anderson.p.jones@gmail.com; 5 Department of Endocrinology, Perth Children’s Hospital, 15 Hospital Avenue, Nedlands 6009, Australia; 6 Institute for Health Research, The University of Notre Dame Australia, Perth 6160, Australia; paola.chivers@nd.edu.au; 7 School of Medical and Health Science, Edith Cowan University, Perth 6027, Australia; 8 The ORIGINS Project, Telethon Kids Institute and Division of Paediatrics, University of Western Australia, 15 Hospital Avenue, Nedlands 6009, Australia

**Keywords:** allergy prevention, eczema, atopic dermatitis, wheeze, food allergy, allergic rhinoconjunctivitis, allergen sensitisation, early childhood, sunlight

## Abstract

The dramatic rise in allergic disease has occurred in tandem with recent environmental changes and increasing indoor lifestyle culture. While multifactorial, one consistent allergy risk factor has been reduced sunlight exposure. However, vitamin D supplementation studies have been disappointing in preventing allergy, raising possible independent effects of ultraviolet (UV) light exposure. The aim of this study was to examine whether UV light exposure influences the development of allergic disease in early childhood. Direct sunlight exposure (290–380 nm) in early infancy was measured via UV dosimeters. Outdoor exposure, sun protective behaviours, and allergy outcomes were assessed over the first 2.5 years of life with clinical assessment appointments at 3, 6, 12 and 30 months of age. Children with eczema had less (*p* = 0.038) direct UV light exposure between 0–3 months of age (median (IQR) 747 (473–1439) J/m^2^) than children without eczema (median (IQR) 1204 (1717–1843) J/m^2^); and less outdoor exposure time (7 min/day) between 11 a.m. and 3 p.m. compared to children without eczema (20 min/day, *p* = 0.011). These associations were seen independent of vitamin D status, and after adjusting for other potential confounders. Whilst we could not find any associations between direct UV light exposure and other allergic disease outcomes, exposure to UV light appears to be beneficial in reducing the risk of eczema development in early childhood. Further research is required to determine optimal levels of UV light exposure while balancing the potential risks.

## 1. Introduction

The prevalence of allergic disease has dramatically increased over recent decades. Genetic factors certainly play a role in the genesis of allergic diseases, but cannot explain the rapid increase seen over this period. When looking for potential factors driving the increasing risk of allergic disease development, the environmental and lifestyle changes of the modern age are clearly implicated [1,2,3,4]. While previously largely an issue in developed countries, the more recent rise of allergic disease in developing countries adds further evidence of the likely impact of increasing urbanization on disease risk [4,5]. While environmental changes are multifactorial, one consistent factor has been a shift from an outdoor to an increasingly indoor lifestyle culture, with greater reliance on digital technology for work and recreation. This global phenomenon [6] affecting all age groups, including during pregnancy and infancy [7,8,9], has led to reduced levels of UV light exposure. UV light plays a crucial role in vitamin D synthesis with an estimated 90 to 95% of human vitamin D provided by cutaneous synthesis under the influence of UVB light [7]. Hence, it is not unexpected that vitamin D deficiency and insufficiency have become global issues—now afflicting more than one billion people globally [7,10,11,12,13,14], including during the critical periods of development of pregnancy and infancy [14,15,16,17].

Considering the known immunomodulatory properties of vitamin D [18,19], this has been a logical pathway to explain the increasing propensity for allergic disease [20,21,22]. However, while epidemiological associations between sunlight exposure (as a surrogate marker of vitamin D status) seemed promising [23,24,25], randomized controlled trials (RCTs) using oral vitamin D supplementation for allergy prevention during pregnancy [26,27] or infancy [28,29] have been disappointing, suggesting that the protective effects of sunlight may be operating through other pathways. Specifically, these trials found limited or no effect on child allergic disease outcomes. A meta-analysis of the pregnancy supplementation RCTs [30] found no overall effect on risk of early childhood (≤4 years of age) atopic dermatitis (3 trials, n = 1538, with 357 cases, relative risk 0.92 (95% confidence intervals 0.77; 1.11) or recurrent wheeze (4 trials, n = 1781, with 358 cases, relative risk 0.76 (95% confidence intervals 0.54; 1.08). Thus, attempting to increase vitamin D status using vitamin D supplementation in pregnancy or infancy as an allergy prevention strategy has yet to be shown to be beneficial. 

For this reason, there has been growing speculation that the epidemiological associations [23,24,25] between reduced sunlight exposure and increased risk of allergic disease may be mediated, at least in part, through immunological consequences of decreased direct UV light exposure rather than vitamin D alone. Indeed, increasing evidence from the field of photobiology has found that UV light can induce immunomodulative effects [31,32]. Exposure to UV light directly induces epidermal cells in the stratum corneum to produce multiple mediators that modulate cutaneous dendritic cells. In turn, these regulate T cell-dependent responses in the skin-draining lymph nodes, with less induction of effector memory cells, and greater production and increased function of T and B regulatory cells. Skin dendritic cells induce peripheral T regulatory cells which migrate from lymph nodes into circulation and back to UV-irradiated skin where they reduce inflammation associated with skin disease [32,33,34]. Likely mechanisms for immunomodulation also include the release of bioactive molecules, such as urocanic acid, from epidermal cells after direct UV light exposure [35,36]. It has been proposed that cis-urocanic acid modulates the production of immunosuppressive molecules which activate T regulatory cells in the lymph nodes and are released into the systemic circulation to reduce inflammatory responses to antigens [31,32]. This ability for cis-urocanic acid to be systemically immunosuppressive has been supported by a study showing a reduced dermal allergic hypersensitivity response after UV exposure [37].

In this study, considering these potential direct immunomodulatory influences of UV light in the context of early life as a critical time for allergy development [20,21,22,38], we sought to further explore whether early life exposure to UV light had vitamin D-independent effects on early childhood allergy outcomes. Specifically, we aimed to examine whether direct UV light exposure (measured by a personal UV dosimeter) in the first 3 months of life reduces the risk of allergic disease in the first 2.5 years of life, and to explore whether sunlight exposure and sun protecting behaviours in early childhood may influence the development of allergic disease in the first 2.5 years of life. 

## 2. Materials and Methods

### 2.1. Study Design

This is an exploratory analysis of data collected as part of a double-blinded RCT investigating the effect of infant oral vitamin D supplementation for the first 6 months of infancy on allergic disease outcomes. Full details of participant screening and infant outcomes to the end of the intervention period at 6 months of age have been previously published [28]. Infant inclusion criteria were term delivery (>37 weeks of gestation) and a family history (parent and/or sibling) of allergic disease, while exclusion criteria were underlying maternal autoimmune disease or immunodeficiency, and maternal 25-hydroxyvitamin D (25(OH)D) level <50 nmol/L or >100 nmol/L at 36–40 weeks of gestation. Written informed consent was obtained before trial participation and included consent to participate in follow-up clinical assessments at 1 and 2.5 years of age. Follow up results of this RCT have also already been published [29] and revealed that infant oral vitamin D supplementation (400 IU cholecalciferol/day) over the first 6 months of life had no influence on allergic disease outcomes over the first 2.5 years of life. Human Research Ethics Committee approvals were granted by the Princess Margaret Hospital for Children (approval number 1959/EP) and the University of Western Australia (approval number RA/4/1/5566). The trial was registered with the Australian New Zealand Clinical Trials Registry (ACTRN12612000787886).

### 2.2. Direct UV Light Exposure Measurements

UV dosimeters (VioSpor blue line Type II, Biosense, Bornheim, Germany) were issued to a subset of the RCT participants, with the aim to obtain objective, quantitative data on individual infant UV light exposure from 0–3 months of age [39,40,41] and provide data on the total UV light (290–380 nm) exposure dose (in joule per square meter [J/m^2^]). Due to funding constraints, we were unable to provide UV dosimeters to all infants participating in the RCT but allocated them to the first 3–4 infants enrolled per month over the course of two years, to distribute the data collection over the 12 months of each year and over the four seasons (summer, autumn, winter and spring). Compliance with the daily UV dosimeter use was captured on diary cards completed by a parent/guardian.

### 2.3. Sunlight Exposure and Sun Protecting Behaviours

At 3 months, 6 months, 1 year and 2.5 years of age, a parent/guardian of all RCT participants (both those allocated a UV dosimeter and those without) were asked questions to capture details on the child’s outdoor and sunlight exposure during the preceding week. We collected parent-reported details on any sun protective behaviours, including frequency of use of sunscreen (always, sometimes or never) and which sun protection factor (SPF) was used. Questions were based on the sunlight exposure questionnaire by Cargill et al. [42]. Details collected also included the question “What time of the day do you take your baby outdoors” distinguishing between estimated averaged time (in minutes/day) spent in:

1. direct sunlight before 11 a.m., between 11 a.m. and 3 p.m., and after 3 p.m.
2. in shade before 11 a.m., between 11 a.m. and 3 p.m., and after 3 p.m.

Questions also assessed the parent-reported average amount of skin exposed to sunlight by body parts (face and hands, or face, hands and arms, or face, hands, arms and legs). In addition, the skin type of each participant (type I to VI, Fitzpatrick scale [43]) was assessed to estimate the possible variable response of different types of skin to UV light.

### 2.4. Vitamin D Supplementation

Vitamin D supplementation ingested doses during the intervention phase of the RCT for each infant participant were calculated during the first 3 months, and from 3 to 6 months of life. The daily dose of vitamin D (400 IU) of the intervention group was based on parent-documented diary card reporting (completed to assess study product compliance) and monthly standardized questions which were also asked by our research team regarding any oral vitamin D supplement intake independent of the trial product use. In addition, information was also captured regarding medically indicated vitamin D supplementation, as all infants with a 25(OH)D level of < 50 nmol/L at 3 months of age were supplemented with appropriate treatment doses of vitamin D.

### 2.5. Allergic Disease Outcome Assessments

At 3 months, 6 months, 1 year and 2.5 years of age the participating children were assessed by a Paediatric Clinical Immunologist at the Princess Margaret Hospital in Perth, Australia. A structured history and a standardized clinical examination to diagnose any allergic diseases were performed. In addition, written reports regarding any hospital presentations/admissions, previous medical reports/assessments and the use of allergy related medications were considered by the assessing immunologist. At 1 and 2.5 years of age, skin prick testing (SPT) was performed using commercially available standardized allergen extracts to the most common allergens amongst young Australian children, including cow’s milk, hen’s egg, peanut, cashew nut, wheat, tuna, house dust mite (Dermatophagoides pteronyssinus), cat and rye grass pollen on the day of clinical assessment. Sensitization was defined as a positive SPT (wheal ≥ 3 mm above negative control) to at least one of the allergens assessed. The participants were assessed for eczema [44], asthma/wheeze, and food allergy at 3 months, 6 months, 1 year and 2.5 years of age. At 2.5 years of age only, the participants were also assessed for a medical diagnosis of allergic rhinoconjunctivitis. Eczema was defined according to the criteria of Hanifin et al. [45] on medical review or when a typical history was taken of an itchy rash distributed to the facial, extensor or flexural surface of the skin following a chronic or fluctuating course. Eczema severity was assessed using the SCORAD index [46] (skin area affected, intensity and subjective symptoms) at each clinical assessment appointment (at 3, 6, 12 and 30 months of age). IgE-mediated food allergy was defined by a history of an immediate (within 90 min) skin rash (urticaria, erythema), angioedema, gastrointestinal symptoms (abdominal pain, vomiting, diarrhea) and/or irritability associated with or without cardiovascular symptoms (collapse) and/or respiratory symptoms (wheeze, stridor, persistent cough, hoarse voice) following the ingestion of a specific food trigger combined with a matching specific food allergen sensitization. Wheeze was defined by a history of an audible wheeze responding to inhaled beta2-agonists. Asthma was defined in children over one year of age only, with a history of recurrent wheeze responding to inhaled beta2-agonists and/or use of a preventer for symptom control. Allergic rhinoconjunctivitis was defined in children over one year of age only with as a history of sneezing or blocked/ runny nose accompanied by itchy or watery/ red eyes unrelated to an upper respiratory tract infection. When wheeze/asthma or allergic rhinoconjunctivitis was combined with sensitization, it was defined as IgE-associated. 

### 2.6. Statistical Methods

Statistical analysis was performed using IBM SPSS Statistics for Windows, version 26 (IBM Corp., Armonk, NY, USA). Data was examined to be missing at random, with an available case analysis approach utilized and respective participant numbers (n) reported throughout. While a pairwise case-by-case analysis approach resulted in maximizing power for analysis, this also presented fluctuations in participant numbers (n) available for each analysis reported which should be considered when interpreting these results. All tests were two-sided, with a *p* < 0.05 considered statistically significant. Categorical data was described using count and percent (%). Scale data was described using mean (M), standard deviation (SD), median (Md), or interquartile range (IQR) with normality assessed using the Shapiro–Wilk test. Between group differences were examined between dosimeter worn and not worn, and between allergic disease and no allergic disease. For categorical variables, these group differences were compared using Chi-Square with a Fisher’s exact 2-sided *p*-value reported. For continuous variables, these were computed using Mann–Whitney U test (not normally distributed), except for birth weight which was normally distributed and assessed using an independent samples *t*-test. 

For the dosimeter worn only group, a generalized linear model with binomial probability distribution and logit link function was used to examine the influence of direct UV light exposure in the first three months (covariate) on the development of allergic disease within the first 2.5 years of life (dependent variable yes/no), with confounders season of birth, skin type, and vitamin supplementation (yes/no). The odds ratio (OR), 95% Wald confidence intervals (CI) and significance reported, and Bonferroni corrected pairwise comparison were conducted as appropriate. The relationship between direct UV light exposure (measured by UV dosimeter over the first 3 months of life) and eczema severity (measure by SCORAD Score) was investigated using Spearman rho correlation coefficients.

Generalized linear mixed models (GLMM) (binomial distribution with a logit link) were used to examine the changes over the four time points (3 months, 6 months, 1 year and 2.5 years) for each of the binary outcomes (present/absent): eczema, wheeze or any allergic disease (atopy). The models included time as the repeated measure. An unstructured random effect for participants was included for the atopy models only. Fixed effects for confounding variables were examined in individual (unadjusted) models (due to sample size limitations) to identify potential confounding: skin type (due to low numbers Fitzpatrick categories I to VI were these collapsed to pale/normal/dark), season of birth, vitamin D levels, outdoor exposure (minutes), sunscreen use (always/sometimes/never) and skin exposed (face/face, hands, arms/face, hands, arms and legs). The model used an unstructured repeated covariance structure, with a robust estimation for model assumptions. Sequential Bonferroni corrected *p*-values are reported. A final adjusted GLMM model was conducted with all significant confounders identified from the unadjusted models.

## 3. Results

### 3.1. Characteristics of the Study Population

Table 1 summarizes the baseline demographic details comparing the infants who were allocated the UV dosimeters (n = 86) to those who were not (n = 109). There were no significant differences between these groups of infants, apart from an increased prevalence of maternal history of allergic disease in the group who were not allocated a UV dosimeter, and differences in season of birth of the infants allocated a UV dosimeter compared to those who were not. Study participant flow details from randomisation to 2.5 years of age follow-up have been previously published [29].

### 3.2. Clinical Allergic Disease Outcomes

The most common allergic disease in the first 2.5 years of life was eczema, affecting a total of 49.1% (81/164) children. By 2.5 years of age, 22.8% (31/136) of the participants were sensitized to at least one allergen, 16.8% (27/161) had medically diagnosed allergic rhinoconjunctivitis and 43.8% (71/162) had medically diagnosed wheeze (see methods Section 2.5). At one year of age, 6.3% (11/175) children were diagnosed with IgE-mediated food allergy and 5.6% (9/162) at the 2.5 year follow up. Egg allergy was the most common food allergy, affecting 9/175 (5.1%) infants in the first year and as expected this had reduced to 5/162 (3.1%) at 2.5 years of age. 

### 3.3. Association between Direct UV Light Exposure in Early Infancy and Allergic Disease Outcomes in the First 2.5 Years of Life

Non-compliance with use of the UV dosimeter occurred with three infants (none of whom developed eczema within the first 3 months of life), leaving 83 infants with useable UV light exposure data. Median (IQR) UV light exposure measured by the UV dosimeters was 950 (556–1577) J/m^2^ from 0–3 months of age, typically with face, hands and arms exposed. 

Sixteen infants had medically diagnosed eczema between 0 to 6 months of age, and as previously published we found an inverse association between direct UV light exposure and eczema outcome at 6 months of age, but no association with wheeze symptoms [28]. Another 24 new cases of eczema were medically diagnosed between 6 months and 2.5 years of age in the subgroup of participants who wore a UV dosimeters in early infancy. 

**Figure 1:**
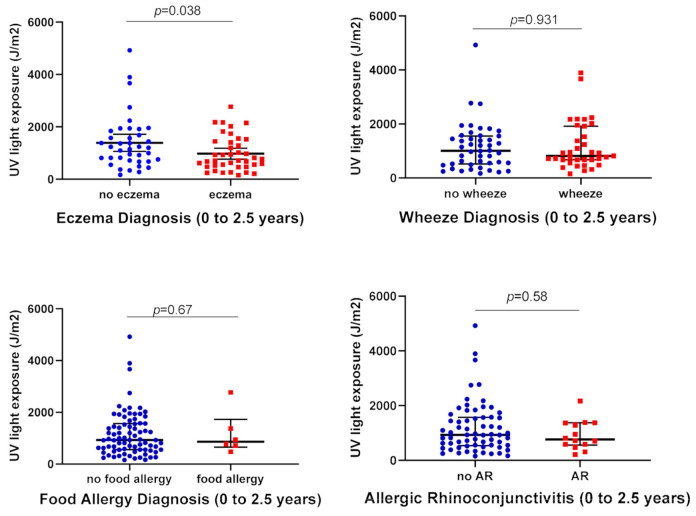
The influence of direct UV light exposure on allergy outcome.

Consistent with previously found eczema outcomes at 6 months of age [28] children with cumulative (birth to 2.5 years of age) medically diagnosed eczema had significantly less direct UV light exposure between 0 to 3 months of age (median (IQR) 747 (473–1439) J/m^2^) than children without eczema (median (IQR) 1204 (1717–1843 J/m^2^) (U -2.07; *p* = 0.038) (Figure 1, Table 2). There were no associations found between UV light exposure levels within the first 3 months of life and eczema severity SCORAD scores at 3 months (r = −0.20, *p* = 0.08), 6 months (r = −0.19, *p* = 0.09), 1 year (r = −0.14, *p* = 0.23) and 2.5 year follow up appointments (r = −0.14, *p* = 0.26).

No significant differences in UV light exposure in early infancy were found between children who had medically diagnosed asthma/wheeze, food allergy or allergic rhinoconjunctivitis, compared to those who did not, within the first 2.5 years of life (Figure 1).

### 3.4. Cross-Sectional Associations between Behaviours Concerning Sun Exposure and Allergic Outcomes within the First 2.5 Years of Life

Total estimated outdoor exposure time by parental report at 3, 6 and 12 months, as well as at 2.5 years of age, was not significantly different between children who developed eczema or any other allergic outcomes within the first 2.5 years of life (Table 2 and Table 3). This also applied for time in the shade or time in the sun exposure in isolation (*p* > 0.05). Examining potential confounding factors at the different time periods such as vitamin D levels, oral vitamin D intake, sunscreen use, exposed skin area, skin type and season of birth, again found no significant differences for cumulative eczema or other allergy outcomes within the first 2.5 years of life (Table 2 and Table 3). 

**Figure 2:**
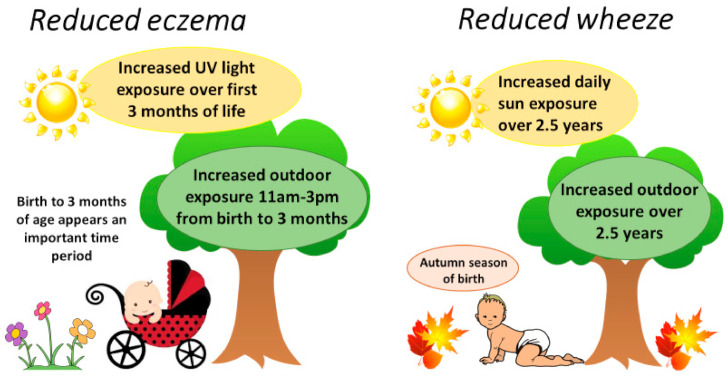
An illustrated summary of the major factors within the first 2.5 years of life that were associated with medically diagnosed eczema and wheeze outcomes.

However, focusing on each specific single time period (time spent outdoors before 11 a.m., between 11 a.m. to 3 p.m., or after 3 p.m.) children with eczema diagnosed over the first 2.5 years of life had significantly less outdoor exposure (n = 79; mean = 7 ± 21.6 min/day) within the first 3 months of life between 11 a.m. and 3 p.m., compared to children without eczema (n = 84; mean = 20 ± 29.1 min/day, *p* = 0.01). Figure 2 includes an illustrated summary of this eczema outcome finding. 

Whereas the group of children developing at least one allergic disease within the first 2.5 years of life spent significantly more time outdoors before 11 a.m. within the first 3 months of life (n = 90, mean = 30 ± 29.9 min/day) than the group who did not develop any allergic diseases (n = 69; mean = 15 ± 31.0 min/day, *p* = 0.03). There were no other significant associations concerning the other time periods spent outdoors (before 11 a.m./between 11 a.m. and 3 p.m./ after 3 p.m.) combined with the participant age groups (0 to 3 months/3 to 6 months/6 to 12 months/1 year to 2.5 years) or specifically time spent in the shade or sun and eczema or other allergic disease outcomes over the first 2.5 years. Again examining potential confounding factors, including vitamin D levels, oral vitamin D intake, sunscreen use, exposed skin, skin type and season of birth, no significant eczema or allergy outcome group differences within the first 2.5 years of age were detected at each of the different time periods. 

### 3.5. Longitudinal Associations between Behaviours Concerning Sun Exposure and Clinical Allergic Outcomes during Early Childhood: Mixed-Effects Logistic Regression Modeling

GLMM for each confounder were examined in individual unadjusted any allergic disease outcome models (any allergic disease development within the first 2.5 years of life). No significant fixed effects were detected for skin type (F = 0.54, *p* = 0.58); season of birth (F = 0.43, *p* = 0.73), or vitamin D levels (F = 3.60 *p* = 0.06). Children that were given vitamin D supplementation for the first 3 months of life (F = 20.6, *p* < 0.001) were at increased risk of any allergic disease (OR = 1.03 CI 1.02–1.04). However, this result did not remain significant after the final GLMM, which included confounding factors. Protective fixed effects included outdoor daily sun exposure time (F = 32.0, *p* < 0.001, OR = 0.98 CI 0.98–0.99), sunscreen use (F = 9.51, *p* < 0.001) for always (OR = 0.38, CI 0.23–0.64, *p* < 0.001) and sometimes (OR = 0.45, CI 0.29–0.68, *p* < 0.001) compared to never. The final GLMM examined any allergic disease with adjustments for confounders and found significant effects for daily outdoor sun exposure, sunscreen and skin exposed (Table 4). 

Individual unadjusted eczema outcome models reported no significant fixed effects detected for skin type (F = 0.60, *p* = 0.55); season of birth (F = 0.20, *p* = 0.90), vitamin D levels (F = 0.12, *p* = 0.73), vitamin D supplementation in the first 3 months (F = 0.05, *p* = 0.82); outdoor exposure (F = 0.10, *p* = 0.75), daily sun exposure (F = 0.15, *p* = 0.70); daily shade exposure (F = 0.001, *p* = 0.98); and skin exposed (F = 0.15, *p* = 0.86). Only sunscreen was a significant confounder (F = 3.41, *p* = 0.03) where children who sometimes wore sunscreen (as opposed to always or never) were at increased risk of eczema (OR = 1.57, CI 1.12–2.20, *p* = 0.009) (Table 4). All children who wore sunscreen used SPF 50.

Wheeze unadjusted models for each confounder were examined with no significant fixed effects detected for skin type (F = 0.49, *p* = 0.61), vitamin D levels (F = 0.18, *p* = 0.67), vitamin D supplementation in the first 3 months (F = 3.8, *p* = 0.05); sunscreen (F = 1.59, *p* = 0.20), skin exposed (F = 2.77, *p* = 0.06) or daily shade exposure (F = 2.46, *p* = 0.12). Protective fixed effects included season of birth (F = 3.35, *p* = 0.02) with a decreased risk of wheeze for children born in autumn (OR = 0.39, CI 0.20–0.75, *p* = 0.005), time spent outdoors (F = 9.1, *p* = 0.003, OR = 1.00, CI 0.99–1.00) and increased daily sun exposure (F = 8.18, *p* = 0.004, OR = 0.91, CI 0.86–0.97). Figure 2 includes an illustrated summary of these wheeze outcome findings. The final adjusted GLMM examined wheeze with confounders season of birth and outdoor exposure, with both remaining significant effects and summarised in Table 4.

Longitudinal modelling was not possible to be conducted for allergic rhinoconjunctivitis and allergen sensitisation, as these were not collected at all four time points; while for food allergy the number of food allergic children was too small to be examined (n = 15 by 2.5 years of age).

## 4. Discussion

This exploratory analysis confirms our previous observations that increased direct UV light exposure in the first three months of life appears to decrease the risk of eczema development [28]. These findings are further supported by parent-reported increased time spent outdoors within the first 3 months of life between 11 a.m. and 3 p.m. (typically the highest UV light exposure time period each day in Australia) with reduced child eczema outcomes over the first 2.5 years of life. In short, the effects of early infancy direct UV light exposure appear to have continuing benefits on reducing eczema development risk into early childhood.

Our findings are supported by cohort studies in the United States [47], Spain [48] and Korea [49], where associations were also observed between eczema prevalence in childhood and mean annual UV indices [47], number of sunny hours in a geographical region [48], or season of birth with estimated UV exposure [49]. A major strength of our study is that we uniquely also measured individual direct UV light exposures using personalized UV dosimeters. This provided objective quantifiable UV light exposure data to correlate with individual child eczema outcomes. Although, due to funding constraints, we were only able to use these personalized UV dosimeters in a sub-set of our trial participants from birth to three months of age, we still found a significant association with early childhood eczema outcomes. We recognize that there was an unbalanced distribution of infants allocated an UV dosimeter according to season of birth. This resulted from variable rates of recruitment in some months each year. We would recommend the use of UV dosimeters (or similar devices) to capture direct individual UV light exposure levels in larger future studies, where extending their use to other time periods during infancy and early childhood should enable further clarification of critical time periods where direct UV light exposure may influence allergic disease outcomes. Future studies would also benefit with the inclusion of genetic profiling of the study participants. For example, examining filaggrin (FLG) gene mutation variants in association with variable UV light exposure levels may further enhance our understanding of this field.

We demonstrated an association of more UV light exposure with reduced eczema outcomes in early childhood, but it remains to be shown as to what extent specifically UVA and/or UVB light is responsible for our observed findings. In this context, it also needs to be considered that our data revealed a longitudinally protective role of sunscreen use for the development of eczema if used sometimes, compared to always when outdoors over the first 2.5 years of life. This finding was unexpected and initially inconsistent with the general understanding that sunscreen protects from UV light penetration. However, it is possible that a specific optimal level or range of UVA and/or UVB light exposure is required to influence specific immune modulation and subsequently allergic disease protection. Thus, further investigations of detailed sunscreen use, including specific quantities (depth and area of skin covered), and specific types of sunscreens used (including composition and presence of any potential allergens, such as peanut/tree nut oils, which potentially could lead to allergen sensitization via a disrupted skin barrier) could be useful in future studies. Important related considerations also include geographical location (latitude) and other influences on UV light penetration like air pollution levels [50].

When examining eczema severity, we did not find a significant inverse association between SCORAD scores and direct UV light exposure measured by UV dosimeter. However, as eczema severity was only measured at the time of clinical assessment, we cannot exclude that the child’s eczema may have been worse at other time points. Eczema severity may have influenced parental decision to take their child outdoors and/or expose them to sunlight, however most children in our study developed eczema after 3 months of age, hence after the period of direct UV light exposure measurement by UV dosimeter.

Our findings, which indicate a vitamin D independent effect of early increased UV light exposure on reduced eczema outcomes, have also been supported in animal models showing that UV radiation leads to antigen-specific-T regulatory cell [51] and dendritic cell expansion causing systemic immunosuppression irrespective of vitamin D [52,53]. Moreover, mast cells [54] and regulatory B-cells affecting dendritic cell mediated T-cell activation, are also involved in UV exposure triggered immunosuppression [55]. A study in mice by Gorman et al. demonstrated that vitamin D was not essential in mediating the immunosuppressive effects of erythema UV-radiation on contact hypersensitivity responses [56]. 

While we found associations with early childhood eczema outcomes, the measurement of direct UV light exposure levels via UV dosimeter failed to show any influence on other early childhood allergy outcomes. However, as this was an exploratory analysis of data collected as part of a double-blinded RCT investigating the effect of infant oral vitamin D supplementation for the first 6 months of infancy on allergic disease outcomes, and not the primary aim of the RCT, this UV light exposure levels analysis was likely underpowered to assess associations with other early childhood allergy outcomes. 

Interestingly, our longitudinal data analysis also revealed a protective link between parent-reported time spent outdoors (specifically time spent in the sun) on wheeze outcomes. Previous cross-sectional observational studies have described an inverse association between vitamin D status and wheeze outcomes or atopy in childhood [57,58,59,60] and longitudinally this finding was also supported by Hollams et al. showing that over a 10-year period, the number of times a child was found to be deficient in vitamin D was positively associated with the risk for eczema, sensitization, asthma and wheeze at age 10 years [61]. However, none of these previous studies evaluated UV light exposure in addition to vitamin D status. Hence, while not specifically examined in these previous studies, it remains possible that vitamin D independent UV-light induced effects may be responsible for their associations with wheeze outcomes.

It is important to remain mindful of the harmful effects of UV light exposure when reporting these potential beneficial effects—including skin damage and malignancy. Balancing the benefits and harms of UV light exposure will likely be an active and essential research area of interest for years to come, especially with increasing urbanization and even global pandemics leading to less time spent outdoors.

## 5. Conclusions

Exposure to UV light in early infancy appears to be beneficial in reducing the risk of eczema development in early childhood. The immunomodulatory effects of UV light are also likely to be important for other immune-based diseases across the life course. Further research in large birth cohorts is required to address this, and to determine optimal levels and patterns of sunlight exposure in infancy and early childhood, and how to balance the potential advantages with risk of adverse effects such as skin damage and cancer outcomes.

## Figures and Tables

**Table 1:** Baseline Characteristics: comparing infants who wore the UV dosimeter for the first 3 months (n = 86) to those who did not wear the UV dosimeter (n = 109).

Characteristic	UV Dosimeter not Worn n (%)	UV Dosimeter Worn n (%)	*p*-Value
Mother completed high school	100/108 (92.6)	83/86 (96.5)	0.35
Maternal white race	90/109 (82.6)	70/86 (81.4)	0.96
Maternal allergic disease	90/108 (83.3)	60/86 (69.8)	0.04
Paternal allergic disease	78/108 (72.2)	68/85 (80.0)	0.24
Infant male sex	58/109 (53.2)	46/86 (53.5)	0.99
Infant birth weight (grams)	3438 ± 374	3395 ± 363	0.42 ^a^
Caesarean-section birth	43/109 (39.4)	32/86 (37.2)	0.77
Infant season of birth			<0.001
Summer	13/109 (11.9)	17/86 (19.8)	
Autumn	18/109 (16.5)	26/86 (30.2)	
Winter	24/109 (22.0)	33/86 (38.4)	
Spring	54/109 (49.5)	10/86 (11.6)	
Infant Fitzpatrick Skin Type			0.81
Type I	1/95 (1.1)	1/84 (1.2)	
Type II	27/95 (28.4)	22/84 (26.2)	
Type III	51/95 (53.7)	40/84 (47.6)	
Type IV	13/95 (13.7)	17/84 (20.2)	
Type V and VI	3/95 (3.2)	4/84 (4.8)	
Smoking in home	6/108 (5.6)	8/85 (9.4)	0.40
Pet cat indoors	17/108 (15.7)	15/85 (17.6)	0.85
Pet dog indoors	56/108 (51.9)	51/85 (60.0)	0.31

Note: ^a^ Group difference *t*-test statistic reported.

**Table 2:** Comparison of cumulative eczema outcomes by 2.5 years of age in association to examined key risk factors.

Outcome			No Eczema (n = 84)		Eczema (n = 81)	
		n	Median (IQR)	n	Median (IQR)	*p*-Value
UV exposure 0–3 months (J/m^2^)		39	1204.0 (717–1843)	40	747.0 (473–1439)	0.038
25(OH)D blood	3 months	65	71.0 (58–90)	62	72.5 (55–88)	0.75
levels (nmol/L)	6 months	69	89.0 (75–106)	63	87.0 (65–112)	0.61
	12 months	73	78.0 (66–92)	70	78.0 (62–94)	0.80
	30 months	68	75.0 (65–88)	56	74.0 (60–88)	0.77
Total outdoor exposure	3 months	84	75.0 (50–116)	79	65.0 (42–105)	0.41
time (minutes/day)	6 months	83	66.0 (40–115)	76	75.0 (41–120)	0.92
	12 months	81	100.0 (65–150)	74	90.0 (60–125)	0.48
	30 months	83	150.0 (105–225)	75	160.0 (120–210)	0.50
			**n (%)**		**n (%)**	***p*-value**
Sunscreen used (3 months)	Always		2/84 (2.4)		3/79 (3.8)	
Sometimes			22/84 (26.2)		14/79 (17.7)	0.46 ^a^
Never			60/84 (71.4)		62/79 (78.5)	
Sunscreen used (6 months)	Always		10/83 (12.0)		8/78 (10.3)	
Sometimes			41/83 (49.4)		29/78 (37.2)	0.21
Never			32/83 (38.6)		41/78 (52.6)	
Sunscreen used (12 months)	Always		14/81 (17.3)		14/78 (17.9)	
Sometimes			52/81 (64.2)		41/78 (52.6)	0.24
Never			15/81 (18.5)		23/78 (29.5)	
Sunscreen used (30 months)	Always		29/84 (34.5)		28/76 (36.8)	
Sometimes			53/84 (63.1)		44/76 (57.9)	0.58
Never			2/84 (2.4)		4/76 (5.3)	
Skin exposed (3 months)	Face		34/84 (40.5)		37/79 (46.8)	
Face, hands and arms			10/84 (11.9)		8/79 (10.1)	0.73
Face, hands, arms and legs			40/84 (47.6)		34/79 (43.0)	
Skin exposed (6 months)	Face		32/83 (38.6)		26/78 (33.3)	
Face, hands and arms			12/83 (14.5)		9/78 (11.5)	0.58
Face, hands, arms and legs			39/83 (47.0)		43/78 (55.1)	
Skin exposed (12 months)	Face		27/80 (33.8)		25/78 (32.1)	
Face, hands and arms			22/80 (27.5)		19/78 (24.4)	0.84
Face, hands, arms and legs			31/80 (38.8)		34/78 (43.6)	
Skin exposed (30 months)	Face		7/84 (8.3)		10/76 (13.2)	
Face, hands and arms			37/84 (44.0)		35/76 (46.1)	0.51
Face, hands, arms and legs			40/84 (47.6)		31/76 (40.8)	
Season of birth	summer		13/84 (15.5)		15/81 (18.5)	
autumn			19/84 (22.6)		18/81 (22.2)	0.89
winter			23/84 (27.4)		24/81 (29.6)	
spring			29/84 (34.5)		24/81 (29.6)	

Note: ^a^ Fisher’s Exact Chi-Square test statistic reported as cell count less than five.

**Table 3:** Comparison of cumulative allergic outcomes within first 2.5 years of life in association to key risk factors.

Outcome			Non-Allergic (n = 69)		Allergic (n = 92)	
		n	Median (IQR)	n	Median (IQR)	*p*-Value
UV exposure 0–3 months (J/m^2^)		32	1174.5 (581–1818)	46	812.5 (511–1380)	0.11
25(OH)D blood	3 months	51	67.0 (56–83)	73	73.0 (57–91)	0.24
levels (nmol/L)	6 months	54	89.5 (74–106)	75	86.0 (67–107)	0.50
	12 months	60	78.0 (66–92)	79	78.0 (61–92)	0.99
	30 months	52	74.5 (62–87)	72	75.0 (65–89)	0.44
Total outdoor exposure	3 months	69	65.0 45–93)	90	70.0 (50–111)	0.63
time (minutes/day)	6 months	67	70.0 (40–125)	88	75.0 (46–104)	0.68
	12 months	66	105.0 (65–166)	85	90.0 (63–125)	0.33
	30 months	68	150.0 (113–210)	91	150.0 (120–240)	0.64
		**n (%)**		**n (%)**		***p*-value**
Sunscreen (3 months)	Always	0/69 (0.0)		5/90 (5.6)		
	Sometimes	16/69 (23.2)		18/90 (20.0)		0.13
	Never	53/69 (76.8)		67/90 (74.4)		
Sunscreen (6 months)	Always	6/68 (8.8)		11/89 (12.4)		
	Sometimes	32/68 (47.1)		37/89 (41.6)		0.74
	Never	30/68 (44.1)		41/89 (46.1)		
Sunscreen (12 months)	Always	10/67 (14.9)		17/88 (19.3)		
	Sometimes	46/67 (68.7)		47/88 (53.4)		0.16
	Never	11/67 (16.4)		24/88 (27.3)		
Sunscreen (30 months)	Always	24/69 (34.8)		34/92 (37.0)		
	Sometimes	43/69 (62.3)		54/92 (58.7)		0.84
	Never	2/69 (2.9)		4/92 (4.3)		
Skin exposed (3 months)	Face		29/69 (42.0)		42/90 (46.7)	
Face, hands and arms			8/69 (11.6)		8/90 (8.9)	0.78
Face, hands, arms + legs			32/69 (46.4)		40/90 (44.4)	
Skin exposed (6 months)	Face		25/68 (36.8)		32/89 (36.0)	
Face, hands and arms			10/68 (14.7)		11/89 (12.4)	0.88
Face, hands, arms + legs			33/68 (48.5)		46/89 (51.7)	
Skin exposed (12 months)	Face		26/66 (39.4)		27/88 (30.7)	
Face, hands and arms			17/66 (25.8)		21/88 (23.9)	0.39
Face, hands, arms + legs			23/66 (34.8)		40/88 (45.5)	
Skin exposed (30 months)	Face		7/69 (10.1)		11/92 (12.0)	
Face, hands and arms			30/69 (43.5)		43/92 (46.7)	0.85
Face, hands, arms + legs			32/69 (46.4)		38/92 (41.3)	
Season of birth	summer		10/69 (14.5)		16/92 (17.4)	
	autumn		16/69 (23.2)		21/92 (22.8)	0.92
	winter		21/69 (30.4)		24/92 (26.1)	
	spring		22/69 (31.9)		31/92 (33.7)	

**Table 4:** Generalised Linear Mixed Models (binary logit link function) for outcomes any allergic disease, eczema and wheeze.

Model Term	*p*-Value	OR	95% CI for OR	
			Lower	Upper
Any Allergic Disease Model				
Daily sun exposure	<0.001	0.987	0.981	0.992
Sunscreen always ^a^	0.05	0.494	0.244	1.000
Sunscreen sometimes ^a^	0.02	0.499	0.279	0.892
Skin exposed face ^b^	0.06	0.589	0.339	1.025
Skin exposed face, hands and arms ^b^	0.01	0.469	0.261	0.841
Vitamin D supplementation	0.11	0.992	0.983	1.002
Eczema Model				
Sunscreen always ^a^	0.40	1.239	0.752	2.039
Sunscreen sometimes ^a^	0.009	1.566	1.117	2.196
Wheeze Model				
Season of birth summer ^c^	0.64	1.197	0.569	2.516
autumn ^c^	0.009	0.417	0.217	0.800
winter ^c^	0.49	0.810	0.444	1.478
Outdoor exposure	0.005	0.996	0.993	0.999

Note: OR odds ratio; ^a^ compared to never; ^b^ compared to face, hands, arms and legs; ^c^ compared to spring.

## Data Availability

The data presented in this study are available on request from the corresponding author. The data are not publicly available due to privacy restrictions.

## References

[1] Okada H., Kuhn C., Feillet H., Bach J.-F. (2010). The ‘hygiene hypothesis’ for autoimmune and allergic diseases: An update. Clin. Exp. Immunol..

[2] Prescott S., Allen K.J. (2011). Food allergy: Riding the second wave of the allergy epidemic. Pediatr. Allergy Immunol..

[3] Nurmatov U., Devereux G., Sheikh A. (2011). Nutrients and foods for the primary prevention of asthma and allergy: Systematic review and meta-analysis. J. Allergy Clin. Immunol..

[4] D’Amato G., Holgate S.T., Pawankar R., Ledford D.K., Cecchi L., Al-Ahmad M., Al-Enezi F., Al-Muhsen S., Ansotegui I., Baena-Cagnani C.E. (2015). Meteorological conditions, climate change, new emerging factors, and asthma and related allergic disorders. A statement of the World Allergy Organization. World Allergy Organ. J..

[5] Odhiambo J.A., Williams H.C., Clayton T.O., Robertson C.F., Asher M.I. (2009). Global variations in prevalence of eczema symptoms in children from ISAAC Phase Three. J. Allergy Clin. Immunol..

[6] Farrag N.S., Cheskin L.J., Farag M.K. (2017). A systematic review of childhood obesity in the Middle East and North Africa (MENA) region: Prevalence and risk factors meta-analysis. Adv. Pediatr. Res..

[7] Holick M.F. (2007). Vitamin D Deficiency. N. Engl. J. Med..

[8] Saraf R., Morton S.M., Camargo C.A., Grant C.C. (2015). Global summary of maternal and newborn vitamin D status—A systematic review. Matern. Child Nutr..

[9] Tanvig M.H., Jensen D.M., Andersen M.S., Ovesen P.G., Jørgensen J.S., Vinter C.A. (2020). Vitamin D levels were significantly higher during and after lifestyle intervention in pregnancy: A randomized controlled trial. Acta Obstet. Gynecol. Scand..

[10] Holick M.F., Chen T.C. (2008). Vitamin D deficiency: A worldwide problem with health consequences. Am. J. Clin. Nutr..

[11] Holick M.F., Siris E.S., Binkley N., Beard M.K., Khan A., Katzer J.T., Petruschke R.A., Chen E., De Papp A.E. (2005). Prevalence of Vitamin D Inadequacy among Postmenopausal North American Women Receiving Osteoporosis Therapy. J. Clin. Endocrinol. Metab..

[12] Marwaha R.K., Tandon N., Reddy D.R.H.K., Aggarwal R., Singh R., Sawhney R.C., Saluja B., Ganie M.A., Singh S. (2005). Vitamin D and bone mineral density status of healthy schoolchildren in northern India. Am. J. Clin. Nutr..

[13] Wagner C.L., Greer F.R., American Academy of Pediatrics Section on Breastfeeding, American Academy of Pediatrics Committee on Nutrition (2008). Prevention of Rickets and Vitamin D Deficiency in Infants, Children, and Adolescents. Pediatrics.

[14] Jones A.P., Rueter K., Siafarikas A., Lim E.-M., Prescott S.L., Palmer D.J. (2016). 25-hydroxyvitamin D status of pregnant women is associated with the use of antenatal vitamin supplements and ambient ultraviolet radiation. J. Dev. Orig. Health Dis..

[15] Hollis B.W., Wagner C.L. (2004). Vitamin D requirements during lactation: High-dose maternal supplementation as therapy to prevent hypovitaminosis D for both the mother and the nursing infant. Am. J. Clin. Nutr..

[16] Lee J.M., Smith J.R., Philipp B.L., Chen T.C., Mathieu J., Holick M. (2007). Vitamin D Deficiency in a Healthy Group of Mothers and Newborn Infants. Clin. Pediatr..

[17] Hossein-Nezhad A., Holick M.F. (2013). Vitamin D for Health: A Global Perspective. Mayo Clin. Proc..

[18] Jeffery L.E., Burke F., Mura M., Zheng Y., Qureshi O.S., Hewison M., Walker L.S.K., Lammas D.A., Raza K., Sansom D.M. (2009). 1.25-Dihydroxyvitamin D3 and IL-2 Combine to Inhibit T Cell Production of Inflammatory Cytokines and Promote Development of Regulatory T Cells Expressing CTLA-4 and FoxP3. J. Immunol..

[19] Jeffery L.E., Wood A.M., Qureshi O.S., Hou T.Z., Gardner D.G., Briggs Z., Kaur S., Raza K., Sansom D.M. (2012). Availability of 25-Hydroxyvitamin D3to APCs Controls the Balance between Regulatory and Inflammatory T Cell Responses. J. Immunol..

[20] Macaubas C., de Klerk N., Holt B., Wee C., Kendall G., Firth M., Sly P., Holt P. (2003). Association between antenatal cytokine production and the development of atopy and asthma at age 6 years. Lancet.

[21] Schaub B., Liu J., Höppler S., Haug S., Sattler C., Lluis A., Illi S., Von Mutius E. (2008). Impairment of T-regulatory cells in cord blood of atopic mothers. J. Allergy Clin. Immunol..

[22] Prescott S.L. (2013). Early-life environmental determinants of allergic diseases and the wider pandemic of inflammatory noncommunicable diseases. J. Allergy Clin. Immunol..

[23] Camargo C.A., Clark S., Kaplan M.S., Lieberman P., Wood R.A. (2007). Regional differences in EpiPen prescriptions in the United States: The potential role of vitamin D. J. Allergy Clin. Immunol..

[24] Mullins R.J., Camargo C.A. (2012). Latitude, Sunlight, Vitamin D, and Childhood Food Allergy/Anaphylaxis. Curr. Allergy Asthma Rep..

[25] Osborne N.J., Ukoumunne O.C., Wake M., Allen K.J. (2012). Prevalence of eczema and food allergy is associated with latitude in Australia. J. Allergy Clin. Immunol..

[26] Chawes B.L., Bønnelykke K., Stokholm J., Vissing N.H., Bjarnadóttir E., Schoos A.-M.M., Wolsk H.M., Pedersen T.M., Vinding R.K., Thorsteinsdóttir S. (2016). Effect of Vitamin D3Supplementation During Pregnancy on Risk of Persistent Wheeze in the Offspring. JAMA.

[27] Litonjua A.A., Carey V.J., Laranjo N., Harshfield B.J., McElrath T.F., O’Connor G.T., Sandel M., Iverson R.E., Lee-Paritz A., Strunk R.C. (2016). Effect of Prenatal Supplementation with Vitamin D on Asthma or Recurrent Wheezing in Offspring by Age 3 Years. JAMA.

[28] Rueter K., Jones A.P., Siafarikas A., Lim E.-M., Bear N., Noakes P.S., Prescott S.L., Palmer D.J. (2019). Direct infant UV light exposure is associated with eczema and immune development. J. Allergy Clin. Immunol..

[29] Rueter K., Jones A.P., Siafarikas A., Lim E.-M., Prescott S.L., Palmer D.J. (2020). In “High-Risk” Infants with Sufficient Vitamin D Status at Birth, Infant Vitamin D Supplementation Had No Effect on Allergy Outcomes: A Randomized Controlled Trial. Nutrients.

[30] Garcia-Larsen V., Ierodiakonou D., Jarrold K., Cunha S., Chivinge J., Robinson Z., Geoghegan N., Ruparelia A., Devani P., Trivella M. (2018). Diet during pregnancy and infancy and risk of allergic or autoimmune disease: A systematic review and meta-analysis. PLoS Med..

[31] Hart P.H., Gorman S. (2013). Exposure to UV Wavelengths in Sunlight Suppresses Immunity. To What Extent is UV-induced Vitamin D3 the Mediator Responsible?. Clin. Biochem. Rev..

[32] Hart P.H., Gorman S., Finlay-Jones J.J. (2011). Modulation of the immune system by UV radiation: More than just the effects of vitamin D?. Nat. Rev. Immunol..

[33] Milliken S.V., Wassall H., Lewis B.J., Logie J., Barker R.N., Macdonald H., Vickers M.A., Ormerod A.D. (2012). Effects of ultraviolet light on human serum 25-hydroxyvitamin D and systemic immune function. J. Allergy Clin. Immunol..

[34] van der Aar A.M., Sibiryak D.S., Bakdash G., van Capel T.M., van der Kleij H.P., Opstelten D.-J.E., Teunissen M.B., Kapsenberg M.L., de Jong E.C. (2011). Vitamin D3 targets epidermal and dermal dendritic cells for induction of distinct regulatory T cells. J. Allergy Clin. Immunol..

[35] Holán V., Kuffová L., Zajícová A., Krulová M., Filipec M., Holler P., Jancárek A. (1998). Urocanic acid enhances IL-10 production in activated CD4+ T cells. J. Immunol..

[36] Sleijffers A., Kammeyer A., de Gruijl F.R., Boland G.J., van Hattum J., van Vloten W.A., van Loveren H., Teunissen M.B.M., Garssen J. (2003). Epidermal cis-urocanic acid levels correlate with lower specific cellular immune responses after hepatitis B vaccination of ultraviolet B-exposed humans. Photochem. Photobiol..

[37] Dahl M.V., McEwen G.N., Katz H.I. (2010). Urocanic acid suppresses induction of immunity in human skin. Photodermatol. Photoimmunol. Photomed..

[38] Metcalfe J.R., Marsh J.A., D’Vaz N., Geddes D.T., Lai C.T., Prescott S.L., Palmer D.J. (2016). Effects of maternal dietary egg intake during early lactation on human milk ovalbumin concentration: A randomized controlled trial. Clin. Exp. Allergy.

[39] Bousquet J., Anto J.M., Demoly P., Schünemann H.J., Togias A., Akdis M., Auffray C., Bachert C., Bieber T., WHO Collaborating Center for Asthma and Rhinitis (2012). Severe Chronic Allergic (and Related) Diseases: A Uniform Approach—A MeDALL—GA2LEN—ARIA Position Paper. Int. Arch. Allergy Immunol..

[40] Siafarikas A., Piazena H., Feister U., Bulsara M.K., Meffert H., Hesse V. (2010). Randomised controlled trial analysing supplementation with 250 versus 500 units of vitamin D3, sun exposure and surrounding factors in breastfed infants. Arch. Dis. Child..

[41] Moehrle M., Korn M., Garbe C. (2000). Bacillus subtilis spore film dosimeters in personal dosimetry for occupational solar ultraviolet exposure. Int. Arch. Occup. Environ. Health.

[42] Cargill J., Lucas R.M., Gies P., King K., Swaminathan A., Allen M.W., Banks E. (2013). Validation of Brief Questionnaire Measures of Sun Exposure and Skin Pigmentation Against Detailed and Objective Measures Including Vitamin D Status. Photochem. Photobiol..

[43] Fitzpatrick T.B. (1988). The validity and practicality of sun-reactive skin types I through VI. Arch. Dermatol..

[44] Hanifin J.M., Cooper K.D., Ho V.C., Kang S., Krafchik B.R., Margolis D.J., Schachner L.A., Sidbury R., Whitmore S.E., Sieck C.K. (2004). Guidelines of care for atopic dermatitis. J. Am. Acad. Dermatol..

[45] Hanifin J.M. (2004). Atopic dermatitis: Broadening the perspective. J. Am. Acad. Dermatol..

[46] Kunz B., Oranje A., Labrèze L., Stalder J.-F., Ring J., Taïeb A. (1997). Clinical Validation and Guidelines for the SCORAD Index: Consensus Report of the European Task Force on Atopic Dermatitis. Dermatology.

[47] Silverberg J.I., Hanifin J., Simpson E.L. (2013). Climatic Factors Are Associated with Childhood Eczema Prevalence in the United States. J. Investig. Dermatol..

[48] Suárez-Varela M.M., Alvarez L.G.-M., Kogan M.D., González A.L., Gimeno A.M., Ontoso I.A., Díaz C.G., Pena A.A., Aurrecoechea B.D., Monge R.M.B. (2008). Climate and prevalence of atopic eczema in 6- to 7-year-old school children in Spain. ISAAC PhASE III. Int. J. Biometeorol..

[49] Hwang J.M., Oh S.H., Shin M.Y. (2016). The relationships among birth season, sunlight exposure during infancy, and allergic disease. Korean J. Pediatr..

[50] D’Amato G., Akdis C.A. (2020). Global warming, climate change, air pollution and allergies. Allergy.

[51] Yamazaki S., Nishioka A., Kasuya S., Ohkura N., Hemmi H., Kaisho T., Taguchi O., Sakaguchi S., Morita A. (2014). Homeostasis of Thymus-Derived Foxp3+ Regulatory T Cells Is Controlled by Ultraviolet B Exposure in the Skin. J. Immunol..

[52] Schwarz A., Navid F., Sparwasser T., Clausen B.E., Schwarz T. (2012). 1,25-Dihydroxyvitamin D Exerts Similar Immunosuppressive Effects as UVR but Is Dispensable for Local UVR-Induced Immunosuppression. J. Investig. Dermatol..

[53] Gorman S., Hart P.H. (2012). The current state of play of rodent models to study the role of vitamin D in UV-induced immunomodulation. Photochem. Photobiol. Sci..

[54] Byrne S.N., Limón-Flores A.Y., Ullrich S.E. (2008). Mast cell migration from the skin to the draining lymph nodes upon UV-irradiation represents a key step in the induction of immune suppression1. J. Immunol..

[55] Byrne S.N., Halliday G.M. (2005). B Cells Activated in Lymph Nodes in Response to Ultraviolet Irradiation or by Interleukin-10 Inhibit Dendritic Cell Induction of Immunity. J. Investig. Dermatol..

[56] Gorman S., Scott N.M., Tan D.H.W., Weeden C.E., Tuckey R.C., Bisley J.L., Grimbaldeston M.A., Hart P.H. (2012). Acute Erythemal Ultraviolet Radiation Causes Systemic Immunosuppression in the Absence of Increased 25-Hydroxyvitamin D3 Levels in Male Mice. PLoS ONE.

[57] Bener A., Ehlayel M.S., Bener H.Z., Hamid Q. (2014). The impact of Vitamin D deficiency on asthma, allergic rhinitis and wheezing in children: An emerging public health problem. J. Fam. Community Med..

[58] Alyasin S., Momen T., Kashef S., Alipour A., Amin R. (2011). The Relationship Between Serum 25 Hydroxy Vitamin D Levels and Asthma in Children. Allergy Asthma Immunol. Res..

[59] Freishtat R.J., Iqbal S.F., Pillai D.K., Klein C.J., Ryan L.M., Benton A.S., Teach S.J. (2010). High Prevalence of Vitamin D Deficiency among Inner-City African American Youth with Asthma in Washington, DC. J. Pediatr..

[60] Baek J.H., Shin Y.H., Chung I.H., Kim H.J., Yoo E.-G., Yoon J.W., Jee H.M., Chang Y.E., Han M.Y. (2014). The Link between Serum Vitamin D Level, Sensitization to Food Allergens, and the Severity of Atopic Dermatitis in Infancy. J. Pediatr..

[61] Hollams E.M., Teo S.M., Kusel M., Holt B.J., Holt K.E., Inouye M., De Klerk N.H., Zhang G., Sly P.D., Hart P.H. (2017). Vitamin D over the first decade and susceptibility to childhood allergy and asthma. J. Allergy Clin. Immunol..

